# Iron Depletion, Iron Deficiency, and Iron Deficiency Anaemia Among Children Under 5 Years Old in Kilimanjaro, Northern Tanzania: A Hospital-Based Cross-Sectional Study

**DOI:** 10.24248/EAHRJ-D-18-00017

**Published:** 2019-07-30

**Authors:** Jonas P Kessy, Rune N Philemon, Abdul Lukambagire, Mwanaidi Abdulrahmani, Glory Urio, Godian Beyanga, Augustine Musyoka, Arnord Ndaro, Ronald Mwitalemi, Maria Maro, Ester Majaliwa, Grace D Kinabo, Blandina T Mmbaga

**Affiliations:** a Department of Paediatrics and Child Health, Kilimanjaro Christian Medical Centre, Moshi, Tanzania; b Kilimanjaro Christian Medical University College, Moshi, Tanzania; c Kilimanjaro Clinical Research Institute, Moshi, Tanzania

## Abstract

**Background:**

Iron depletion results from reduced iron stores, and it is an early stage of disease progression before iron deficiency, which leads to iron deficiency anaemia (IDA). IDA is associated with delayed infant growth and development, diminished cognitive function, poor academic performance, decreased exercise tolerance, and impaired immune function. This study aimed to determine the prevalence of iron depletion and IDA and factors associated with low ferritin levels among children under 5-years-old receiving care at Kilimanjaro Christian Medical Centre (KCMC) in Moshi, Tanzania.

**Methods:**

Under-5 children presenting at KCMC were successively enrolled and screened for iron depletion and IDA using complete blood count and serum ferritin levels. The generally accepted World Health Organization cut-off levels for normal haemoglobin (Hb) and ferritin level were used. Iron depletion, iron deficiency, and IDA prevalences were estimated in relation to the combination measures of haemoglobin, mean corpuscular volume, and ferritin levels. Dietary and sociodemographic characteristic of the children were recorded after parents or caretakers provided informed consent. Data analysis was conducted using SPSS version 21.0.

**Results:**

A total of 303 children aged 2 to 59 months were enrolled in the study. Anaemia was detected in169 (55.8%) children. Children aged 2 to 12 months had a higher prevalence of anaemia (n=101, 60.1%). The overall prevalences of iron depletion, iron deficiency with no anaemia, and IDA were 2.6% (n=8), 9.6% (n=29), and 28.1% (n=84), respectively. Low ferritin levels were detected in 124 (40.9%) children. Drinking more than 500 ml of cow's milk per day was associated with an increased risk of anaemia (adjusted odds ratio [AOR] 5.6; 95% confidence interval [CI], 2.6 to 12.1) relative to those not drinking cow's milk. Children whose families had meals that included beef more than 3 times per week were less likely to have low ferritin (AOR 0.6; 95% CI, 0.3 to 1.3), though the difference was not significant.

**Conclusion:**

The IDA prevalence among children in the Kilimanjaro area was high, with more than 50% of infants being anaemic. Drinking cow's milk was associated with an increased risk of IDA. Future community-based research is recommended to elucidate more details about iron deficiency in the general population.

## INTRODUCTION

Iron depletion results from reduced iron stores and is an early asymptomatic stage of micronutrient deficiency. If not corrected, iron depletion has the potential to progress to iron deficiency and iron deficiency anaemia (IDA). The progression is usually considered in 3 phases; iron depletion (normal haemoglobin, normal Mean corpuscular volume (MCV) but low ferritin level), iron deficiency with no anaemia (normal haemoglobin, low MCV level and low ferritin level) and IDA (low haemoglobin, low MCV and low ferritin levels).^1^ IDA is associated with delayed infant growth and development, diminished cognitive function, poor academic performance, decreased exercise tolerance, and impaired immune function.^2-5^

IDA among children under 5 years of age has a prevalence of 47.4% worldwide and ranges from 18% to 26% among children under 5 years old in developed countries.^6-8^ A recent report, which included several African countries, reported a prevalence of anaemia in children below 5 years old, ranging from 10.5% in South Africa to 75% in Cote d'Ivoire, while IDA ranged from 11% in South Africa to 64% in Egypt.^9^ In Tanzania, the overall prevalence of anaemia was 77.2%, and the prevalence of IDA based on the ferritin level was 22.6%.^10^ Several risk factors have been associated with IDA and low ferritin levels, which include; poverty, being born prematurely, maternal anaemia, intestinal infestations like worms, infections, haematological disorders and nutritional factors like poor eating behaviours and use of cow's milk in children below the age of twelve months.^4,11,12^

There are several different methods for measuring anaemia, including complete blood count (CBC), total iron-binding capacity, serum iron, serum transferrin, and serum ferritin levels which are used either singly or in combination to assess iron levels in patients. Serum ferritin is the major form in which iron is stored in the body, and measurement of this can easily detect early changes in body iron storage, thereby making it the preferred single best blood test for the diagnosis of iron deficiency.^4,13-15^

Despite the high prevalence of IDA (11%-64%) in developing countries,^9,10^ the challenge is lack of resources for early diagnosis for iron depletion and iron deficiency. Iron depletion is asymptomatic and has unclear risk factors, thereby hampering a clinical diagnosis. Early detection would enable early intervention; therefore, knowing the risk factors and magnitude of the problem in a specific setting will allow primary prevention and hence prevent the harmful effects of IDA which are not reversible.

The most common causes of iron deficiency in children include insufficient intake together with rapid growth, low birth weight and gastrointestinal losses related to excessive intake of cow's milk,^16^ and parasites infestation such as helminth.^17^ Therefore, the main management of IDA should include identification and treatment of the underlying cause for IDA, iron replacement, nutritional counselling and advice as well as educational to the parent and family as well as helminthic control through deworming.^16,17^

This study aimed to determine the prevalence of iron depletion and IDA and factors associated with low ferritin levels among under-5 children receiving care at Kilimanjaro Christian Medical Centre (KCMC) paediatric department to inform planning for interventions to prevent IDA in children.

## METHODS

### Study Design and Study Site

This was a cross-sectional, hospital-based study carried out at the paediatric outpatient clinic (POPD), Reproductive and Child Health service (RCHS), Human Immunodeficiency Virus (HIV) exposed clinic and within the paediatric ward from August 2014 to July 2015 at KCMC in Moshi, Tanzania. KCMC is a faith-based organisation located in Kilimanjaro region in the northern part of Tanzania serving a population of approximately 15 million. KCMC has the official bed capacity of 638 beds with 130 beds, specifically being for paediatric care. The hospital receives referrals from the northern zone of Tanzania, and several outpatient clinics are attended daily. Daily, more than 1,000 patients are seen as outpatients, with about 20% being children. Paediatric outpatient clinics include HIV/AIDS clinic known as Child Centred family care clinic (CCFCC), general paediatric known as POPD clinic, neuropaediatric outpatient clinic (NPOC) for children with neurological disorders, paediatric outpatient neonatal follow-up clinic (POPNF) as well as the reproductive and child health services (RCHS) for wellbeing under 5 for vaccination and development. For this study, we included children attending POPD, CCFCC and RCHS.

In the Kilimanjaro region, the main activities remain agricultural production with the main being coffee, banana, maize and beans plantation. The region is divided into 4 different zones which favour the agro-economic activities in the region: (1) the Coffee Zone (coffee, bananas, maize, beans, and dairy production), (2) the Wheat Zone (wheat, beans, maize and dairy production), (3) the Lower Zone (maize, cotton, beans, paddy, and suitable for ranching), and (4) the Forestry Zone, which accounts for 388,500 ha of forest and national park.^18^

### Study Population, Inclusion and Exclusion Criteria

The study population included children aged 2-59 months who attended POPD, CCFCC and RCHS clinics as outpatients or inpatients, whose mothers agreed to sign informed consent. Children who had a history of blood transfusion within 3 months before recruitment, regular iron supplementation for more than 2 months with a known haematological disorder, tuberculosis, HIV/AIDS, history of prematurity, or active haemorrhage were excluded. Information on age, sex, birth weight, residential address, breastfeeding history, use of cow's milk (including amount used), child's intake of meat (including amount).

This study used CBC and serum ferritin level to assess the level of iron storage in the study population using the World Health Organization (WHO) general accepted cut-off levels as; Hb >11mg/dl, MCV >80fl and serum ferritin (SF) level 12 ng/ml, Mild moderate and severe anaemia was considered when Hb level is = 10.0-10.9 mg/dl (Mild), 7.0-9.9 mg/dl (moderate) Less than 7 mg/dl (severe).^19,20^ Iron depletion was defined as a state when Hb >11mg/dl, normal MCV (80fl) and serum ferritin (SF) level is below 12 ng/ml, while iron deficiency is when the Hb is >11mg/dl with low MCV (<80fl) and low SF (<12ng/ml) while IDA is when Hb is <11mg/dl, and MCV <80fl with low SF (<12ng/ml).^6,13,21^

### Sample Size Estimation

The minimum sample size was estimated using a formula by the Survey System Creative Research and Joint WHO (1988) expressed as sample size = Z^2^(p)(1-p)/ε^2^, where, Z = value (1.96 for 95 % confidence level).**** A prevalence (P) of 24 % for IDA reported from the Tanzania demographic and health 2010^22^ and ε = minimal tolerable error at the 95% confidence level, expressed as a decimal (0.05). The minimum estimated sample size was 303 participants. The study used a convenience sampling technique where all children seen at the outpatient and inpatient who fulfilled the inclusion criteria during the study period were enrolled.

### Laboratory Sample Analysis

Venous blood (4 to 5 ml) was drawn where, 2 ml of blood was placed in the EDTA tube for analysis of Complete blood count with Mindray 3200 haematology analyser (Shenzhen Mindray Bio-Medical Electronics Co., Ltd) at the KCMC Clinical laboratory. The remaining 3 ml of blood was placed in a serum separation tube (SST) then transported to Kilimanjaro Christian Research Institute (KCRI) – Biotechnology laboratory, situated within KCMC campus. The KCRI-Biotechnology Laboratory is located on the campus of KCMC, about 500 meters from the Hospital buildings. It supports both clinical trials and basic science research.

Centrifugation was done at 1,000 to 1,300 rpm for 10 minutes. Serum was collected in cryotubes and stored in a refrigerator at −20°C. Samples batched and tested for serum ferritin by ELISA using a commercial kit (Pishlaz Teb Diagnostics Zaman; catalogue no PT –Ferr -96, Germany). Laboratory data sheets were used to record all laboratory results. All children identified with iron depletion, iron deficiency, or IDA were started on iron supplementation and follow up given at the regular paediatric outpatient clinic every 3 months until 6 months after MCV and MCH have normalised.

### Data Analysis

Data analysis was done using SPSS version 21.0 (IBM Corp., Armonk, NY, USA). Descriptive statistics were estimated where frequency count, and percentages were reported. Logistic regression was used to determine factors associated with iron depletion, iron deficiency, and IDA. Chi-square tests were used to test for significance in each of characteristics of the population at *P*≤.05.

### Ethical Considerations

The ethical clearance was obtained from Kilimanjaro Christian Medical University College (KCMUCo) ethical review committee with a certificate No. 711. Informed consent was obtained from the parents/guardian of the study participants before enrollment. To ensure confidentiality, no names were used, and to minimise pain, the smallest needle was used to collect the minimum amount of blood required. Each participant's parent/guardian was informed of the results of haemoglobin level and the implications. Iron supplementation was prescribed when necessary. Children whose parents or guardians refused to consent received the same service provision and care as children who participated in the study.

## RESULTS

We enrolled 314 children aged below 60 months, of which 6 did not show up for sample collection, and the samples from another 4 were insufficient for complete blood count and serum ferritin and 1 sample clotted. Therefore, 303 children samples were obtained and analysed, of which 153 (50.5%) were male and 150 (49.5 %) female. Median (IQR) age in months was 10 (6-18). The majority, (n=169, 55.8%) were in the age group 2-12 months. Two hundred fifty-eight (85.1%) had a normal birth weight between 2.5 and 4.0 kg with a median weight (IQR) at birth of 3.2 kg (2.9-3.5) and 74 (24.4%) of children being exclusively breastfed at the time of the study (Table 1).

**TABLE 1.** Baseline Characteristics (N=303)

Characteristics	n	%
**Sex**		
Male	153	50.5
Female	150	49.5
**Age, months**		
2-12	169	55.8
13-23	78	25.7
24-59	56	18.5
**Median**	10 (IQR: 6-18)	
**Religion**		
Christian	263	86.8
Muslim	40	13.2
**Region of residence**		
Kilimanjaro	283	93.4
Manyara	5	1.7
Arusha	13	4.3
Other	2	0.7
**Birth weight, kg (^a^N=299)**		
<2.5	30	9.9
2.5-4.0	258	85.1
>4	11	3.6
**Median**	3.2 (IQR: 2.9-3.5)	

a Missing data for 4 participants

Abbreviation: IQR, interquartile range

### Prevalence of Iron Depletion, Iron Deficiency, and IDA

Of the 303 participants, 168 (55.5%) had anaemia, of these 79/168 (47.1%) had mild anaemia (Hb 10.0 - 10.9mg/dl) 87/168 (51.7%) had moderate anaemia (Hb 7.0 - 9.9mg/dl) and 2/168 (1.2%) had severe anaemia (Hb Less than 7mg/dl). In total, 84 (28.1%) children had IDA, 29 (9.6%) had iron deficiency with no anaemia, and 8 (2.6%) had iron depletion. A total of 124 (40.9%) children in the study had low ferritin level. Among children with anaemia, the majority (n=101, 60.1%) were aged 2 to 12 months (Table 2).

**TABLE 2.** Prevalence of Anaemia, Iron Depletion, Iron Deficiency With No Anaemia, and Iron Deficiency Anaemia (N=303)

Categories	Total	2-12 months	13-23 months	24-59 months
		n (%)	n (%)	n (%)
**Low Serum ferritin**	124	60 (48.4)	49 (39.5)	15 (12.1)
**Anaemia**	168	101 (60.1)	52 (31.0)	15 (8.9)
**Mild**	79	49 (62.0)	23 (29.1)	7 (8.9)
**Moderate**	87	52 (59.8)	28 (32.2)	8 (8.0)
**Severe**	2	0(0)	1 (50.0)	1 (50.0)
**Iron depletion**	8	4 (50.0)	1 (12.5)	3 (37.5)
**Iron deficiency**	29	11 (37.9)	14 (48.3)	4 (13.8)
**Iron deficiency anaemia**	84	43 (50.6)	34 (40.0)	8 (9.4)

### Factors Associated With Low Ferritin Levels

The factors found to be independently associated with low ferritin levels after adjustment were sex, whereby males had nearly twice the odds of having a low ferritin level (adjusted odds ratio [AOR] 2.0; 95% confidence interval [CI], 1.2 to 3.4), compared to female counterparts. Infants and toddlers had higher odds of having low ferritin levels as compared to pre-school children (AOR 2.7; 95%CI, 1.2 to 6.1 and AOR 5.0; 95% CI, 2.0 to 11.2, respectively) (Table 3). Drinking more than 500 ml of cow's milk per day was associated with increased risk of anaemia (AOR 5.6; 95%CI, 2.6 to 12.1) as compared to children who had not used cow's milk. Children whose family who ate beef 3 or more times per week were less likely to have low ferritin (AOR 0.6; 95% CI, 0.3 to 1.1) as compared to those who had not used meat; however, this was not significant.

**TABLE 3.** Factors Associated With Low Ferritin Level (N=303)

Variable	Ferritin		Unadjusted OR (95% CI)	Adjusted OR^a^ 95% CI)
	Normal n (%)	Low n (%)		
**Sex**				
Male	79 (51.6)	74 (48.4)	1.9 (1.2–3.0)	2.0 (1.2–3.4)
Female	100 (66.7)	50 (33.3)	Ref	Ref
**Age, months**				
2-12 (Infants)	95 (57.2)	71 (42.8)	1.2 (0.7–2.3)	2.7 (1.2–6.1)
13-23 (Toddlers)	47 (60.3)	31 (39.7)	1.1 (0.6–2.2)	5.0 (2.2–11.2)
24-59 (Pre-school)	37 (62.7)	22 (37.3)	Ref	Ref
**Use of cow's milk**				
No	60 (81.1)	14 (18.9)	Ref	Ref
Yes	119 (52.0)	110(48.0)	4.0 (2.1–7.5)	-
**Amount of cow's milk**				
≥500	69 (45.1)	84 (54.9)	5.2 (2.7–10.1)	5.6 (2.6–12.1)
<500	50 (65.8)	26 (34.2)	2.2 (1.1–4.7)	2.2 (1.0–5.0)
No cow's milk	60 (81.1)	14 (18.9)	Ref	Ref
**Inclusion of red meat**				
≥3	38 (65.5)	20 (34.5)	0.9 (0.5–1.7)	0.6 (0.3–1.3)
<3	36 (46.2)	42 (53.8)	2.0 (1.2–3.4)	1.6 (0.7–3.1)
No red meat	105 (62.9)	62 (37.1)	Ref	Ref

a Adjusted for time of weaning, age started cow's milk, birth weight, time of breast feeding and past 2 months medical history

Abbreviations: OR, odds ratio; Ref, reference category

## DISCUSSION

In this study, we aimed to determine the prevalence of iron depletion, IDA, and factors associated with low ferritin level in children aged 2-59 months. The overall prevalence of anaemia in this population was 55.8% with more than 60% of anaemia being amongst aged 2-12 months. The prevalence of iron depletion was 2.6%, iron deficiency with no anaemia 9.6%, and IDA 28.1%. Low ferritin level was observed in 40.9% of the children. Various factors were observed to independently affect low ferritin levels the outcome, including young age, male sex, and drinking cow's milk. This suggests a need for nutritional improvement and educational to the parents.

The anaemia prevalence among children below 5 years of age was high (55.8%). Similar prevalence rates were reported in Brazil (56.6%)^23^ and Nigeria (57.1%).^24^ The prevalence in our study was lower than those reported in Mwanza, Tanzania (72.2%).^10^ The study in Nigeria is the only study which had a similar age range as our study (2 to 59 months) whereas; other studies used the age group from 6-59 months. The differences noted in children from different settings are most likely due to ingestion of food with low iron contents, a higher burden of worm infestations in other settings and less ingestion of micronutrients with higher use of cow's milk. Despite the high prevalence, in our study, only 2 children were reported to have severe anaemia. This low number is similar to what others have reported, suggesting the condition is often picked up before getting to a severe state.

The observed prevalence of iron depletion in our study was low (2.6%), which differed from other studies from Iran (19.7%),^7^ United Arab Emirates (26.4%),^8^ and New Zealand (18.6%).^6^ The prevalence of iron deficiency with no anaemia was 9.6% which is lower than a previous study conducted in Mwanza, Tanzania, where the prevalence was 33.3%.^10^ This difference could be due to regional differences in the type of porridge traditionally used for weaning. In Kilimanjaro mothers typically use finger millet porridge with milk being used for weaning while in Mwanza, maize porridge is more commonly used. Finger millet is a better source of iron and folate when compared to maize. The findings from our study are lower than those in developed countries, (for example 5.6% in New Zealand^6^ and 7% in the USA.^8^ The use of fortified food/milk and the avoidance of cow's milk before the age of 1 year may be the reason for this low prevalence in the developed countries.

The prevalence of IDA in our study (28.1%) was almost similar to the 24%, which was reported previously in the 2010 Tanzania demographic and health survey.^22^ However, the prevalence of IDA was higher when compared to what has been reported in previous studies in other developing countries such as Nigeria 14.9% and United Arab Emirates 9.9%,^8,25^ and developed countries such as USA(8%), New Zealand (4.3%) and Turkey (3.29%).^5,6^ Cow's milk before the age of 1 year is known to be a risk for anaemia.^6,9,26^ In our study, we also observed that children who drink more than 500 ml of cow's milk a day had lower ferritin compared to children who drink less than 500 ml per day. Similar findings were also previously reported in New Zealand.^6^ The lower observed prevalence of IDA compared to a study in Mwanza (37.5%),^10^ this might be as explained by differences in cultural and socioeconomic status, which may impact the type of food eaten as well as milk consumption. Interestingly, the time of weaning was not an independent predictor for iron deficiency or IDA, and the majority of the children less than 12 months in this study were not supplemented with iron. A study by Kadivar et al. in Iran found that iron supplementation in the first year of life appears to be protective to anaemia and iron deficiency.^7^

Low ferritin level was observed in 40.9% of children in this study, which is similar to the 32.6% reported in Mexico.^27^ In this study, female children were less likely to have low ferritin level when compared to males; this was not the case in a study in New Zealand where females were at increased risk of developing anaemia.^6^ Children who had a lower levels of red meat consumption (less than 3 days per week) had a slightly increased risk of having low ferritin levels. This was consistent with the findings from Nigeria, where dietary intake of vegetables and animal products less than 3 times a week was significantly associated with a lower IDA prevalence.^25^ Red meat has a high iron content and is a good source of absorbable haeme iron.

In general, cow's milk has several properties that lower iron uptake, including low iron availability and excessive protein and calcium which inhibit the absorption of iron as reported in a review by Olivera et al,^28^ therefore, iron supplementation should definitively be recommended when using cow's milk to feed infants as not all cow's milk formulae used are fortified with iron.

### Limitations

This was a hospital-based study; therefore, the prevalence of IDA may not truly reflect the general population. It is likely that the prevalence in this study overrepresents that of the general population. A community-based study might give us a better estimation of the actual situation within the community.

## CONCLUSION AND RECOMMENDATIONS

The prevalence of IDA in children in the Kilimanjaro area was high, with more than 50% of infants in this study having IDA. Nutritional counselling on iron-rich food and the increased risk of IDA if cow's milk is used under 12 months of age may help to prevent IDA to under-5 children. Future community-based study is recommended to get the actual situation on iron deficieny in the general population.
